# Cortical Depth Dependent Functional Responses in Humans at 7T: Improved Specificity with 3D GRASE

**DOI:** 10.1371/journal.pone.0060514

**Published:** 2013-03-22

**Authors:** Federico De Martino, Jan Zimmermann, Lars Muckli, Kamil Ugurbil, Essa Yacoub, Rainer Goebel

**Affiliations:** 1 Center for Magnetic Resonance Research, University of Minnesota Medical School, Minneapolis, Minnesota, United States of America; 2 Department of Cognitive Neuroscience, Faculty of Psychology and Neuroscience, Maastricht University, Maastricht, The Netherlands; 3 Maastricht Brain Imaging Center, Maastricht University, Maastricht, The Netherlands; 4 Center for Cognitive Neuroimaging, Department of Psychology, University of Glasgow, Glasgow, United Kingdom; Department of Neuroimaging and Neuromodeling, Netherlands Institute for Neuroscience, an Institute of the Royal Netherlands Academy of Arts and Sciences, Amsterdam, The Netherlands; University of Massachusetts Medical School, United States of America

## Abstract

Ultra high fields (7T and above) allow functional imaging with high contrast-to-noise ratios and improved spatial resolution. This, along with improved hardware and imaging techniques, allow investigating columnar and laminar functional responses. Using gradient-echo (GE) (T2* weighted) based sequences, layer specific responses have been recorded from human (and animal) primary visual areas. However, their increased sensitivity to large surface veins potentially clouds detecting and interpreting layer specific responses. Conversely, spin-echo (SE) (T_2_ weighted) sequences are less sensitive to large veins and have been used to map cortical columns in humans. T_2_ weighted 3D GRASE with inner volume selection provides high isotropic resolution over extended volumes, overcoming some of the many technical limitations of conventional 2D SE-EPI, whereby making layer specific investigations feasible. Further, the demonstration of columnar level specificity with 3D GRASE, despite contributions from both stimulated echoes and conventional T_2_ contrast, has made it an attractive alternative over 2D SE-EPI. Here, we assess the spatial specificity of cortical depth dependent 3D GRASE functional responses in human V1 and hMT by comparing it to GE responses. In doing so we demonstrate that 3D GRASE is less sensitive to contributions from large veins in superficial layers, while showing increased specificity (functional tuning) throughout the cortex compared to GE.

## Introduction

With the advent of high-resolution functional magnetic resonance imaging (fMRI) at high fields, new possibilities have emerged that may pave the way for a better understanding of human brain function. High field high-resolution fMRI studies have permitted investigations into cortical columns, the fundamental unit of neural organizations [1], [2]. They have demonstrated the feasibility of robust columnar mapping in humans, depicting ocular dominance [3]–[8], and orientation columns [9] in V1, as well as columns related to axis of motion in area V5/hMT [10]. The ability to achieve sub-millimeter resolution also permits the noninvasive measurement of hemodynamic responses at different cortical depths, making it feasible to examine the different processing stages within a cortical region as well as layer-specific activity across different brain areas in terms of inputs and outputs [11]. Several studies on laminar specific responses have been reported in animal models using cerebral blood volume (CBV), gradient-echo (GE), or spin-echo (SE) weighted BOLD fMRI [12]–[20]. In humans there have been a few layer specific studies, all of which used GE BOLD [21]–[24] except for our group's most recent work [25].

fMRI at high magnetic fields benefits from increased BOLD-based susceptibility contrast [26], [27] as well higher signal-to-noise ratios (SNR) [28], [29]. It is this resulting increase in the functional contrast-to-noise ratio (CNR) that permits the acquisition of higher spatial resolution images. With increasing spatial resolutions, partial volume effects are reduced, and the proportion of voxels that uniquely sample (sections of) the gray matter increases. However, higher spatial resolution alone is not sufficient to assure higher spatial specificity, as the BOLD response is ultimately limited by the underlying vascular contributions and the spatial point spread function of these contributions. Standard gradient-echo (T_2_*) based BOLD contrast, which is used in the vast majority of fMRI studies, is sensitive to both micro and macro-vasculature components at high magnetic fields [12], [13], [15], [30]–[32] and is influenced by both oxygenation related vascular changes as well as large draining effects from vessels penetrating the cortex orthogonally or from pial veins lying on top of the gray matter surface. Capillary density usually shows a rough correspondence to the cortical layer structure derived from cytoarchitecture. The layer exhibiting the highest capillary density is arranged at about the same location as neuronal layer IV of the cortex (in those regions where this laminar arrangement is present), while each cortical layer is additionally penetrated vertically by a fine network of diving venules/terminal arterioles and horizontally by arterioles connected to feeding arteries and large draining veins across the pial surface [33]–[35]. It has been shown that mapping columns or functional patterns running orthogonal to the cortical surface is more reliably achieved with SE based BOLD fMRI, which is less sensitive to these surface vein effects [8], [36]. Cortical depth dependent fMRI studies in animals and humans [12]–[15], [21]–[24] using standard gradient echo EPI have shown a steep increase in signal change near and beyond the CSF border of the cortex using both standard and higher field magnets. This large pial surface effect may compromise the spatial specificity of columnar or layer specific fMRI mapping via partial volume effects or extravascular spreading of the BOLD response some distance away from the surface. The relative magnitude of GE BOLD signals near the middle of the cortex is unclear from the literature. The finding of an increased response in cortical layer IV compared to neighboring layers [21], [23], [24], has not always been observed in human studies [22]. However, this single condition activation profile does not directly suggest anything about the specificity of the response as would be obtained from a differential paradigm. Further, whether the use of SE based sequences at high fields, which can significantly reduce extravascular BOLD signals from surface veins spreading over large distances, would provide any advantages over GE acquisitions for layer specific studies is unclear.

While T_2_ weighted sequences can show greatly increased spatial accuracy, they come with the drawback of higher radiofrequency (RF) power deposition resulting from 180° refocusing pulses, the sensitivity to non-uniform B_1_ fields, reduced BOLD contrast [30], [31], and ultimately significantly reduced volume coverage compared to conventional GE based methods. Also, for SE-EPI based acquisitions, the hypothesized reduced sensitivity to large superficial draining veins has been shown to be dependent on the T_2_* contribution inherent in the EPI readout and enhanced with longer echo train lengths [14]. Because of the aforementioned limitations, the successful application of SE-EPI for human high-resolution (columnar) fMRI has been limited to a single thick slice and with an extremely small FOV [8], [9]. Such an approach is impractical for applications outside of primary visual areas and for those requiring high isotropic resolutions, such as the study of cortical layers. Until our group's most recent work [10], [25] using a 3D GRASE (gradient and spin echo) sequence, no other alternatives to conventional 2D SE-EPI have demonstrated the ability to achieve high resolution T_2_ weighted functional images in humans and there have been no layer specific SE fMRI studies in humans. Using 3D GRASE imaging [37], [38] with a train of refocused pulses to encode a 3D slab combined with inner volume excitation, facilitates the efficient acquisition of high isotropic resolution images with higher SNR over extended volumes compared to inner volume based 2D SE-EPI, which has produced high resolution fMRI maps in humans. The resulting 3D images, however, do have stimulated-echo weighted contrast [39] in addition to T_2_ weighting. Despite this, we were able to successfully map axis of motion columns in human area MT [10], demonstrating the high degree of spatial specificity achievable with 3D GRASE.

This study set out to investigate the characteristics of cortical depth dependent fMRI signals at ultra-high field (7T) and whether or not 3D GRASE acquisitions can provide any specificity advantages over conventional GE-EPI, the current popular choice for cortical depth dependent fMRI studies in humans. We recorded functional responses from two visual areas (V1 and hMT) using both sequences and then used high-resolution cortical grid sampling tools to obtain 3D sampled relative cortical depth layer responses from folded cortical tissue.

## Methods

### Ethics Statement

The institutional review board for human subject research at the University of Minnesota approved all studies. All subjects gave written informed consent before participating in the study.

### Subjects

Four healthy volunteers (1 male, 3 females) with normal or corrected to normal visual acuity participated in the study. The study involved two experiments, the first on the layer/columnar response of human area MT and the second on the layer responses of human area V1. Both experiments comprised two scanning sessions (localizer/GE session and GRASE session). Two of the volunteers participated in both experiments. To reduce the influence of motion, subjects employed a bite bar made out of a hydro plastic material (Tak Systems) that matched their dental impressions.

### Experimental design and stimuli

Visual stimuli were presented using a video projector and mirror at the rear of the magnet. An additional mirror was attached to the coil above the subject's eyes, allowing them to view the screen behind the coil where the image was projected. Stimuli were presented using custom-built stimulation software (StimulGL) for the MT stimuli as well as Presentation (Neurobehavioral Systems, CA, USA) for V1 stimulation. Subjects were instructed to fixate on a small central fixation spot during the entire functional scan. All subjects were trained and experienced at maintaining fixation.

For the MT experiment, during the first session, area hMT was functionally defined, based on its responses to stimuli alternating between moving and stationary dot patterns, which were presented to either the right, left or both visual fields following standard procedures (i.e. human MT+ mapping) [40]–[42]. Left, right and both lateral localization stimuli and their static counterparts were presented 8 times, blocked into two functional runs of 5 min and 52 s each (176 volumes per run). We also mapped axis of motion direction preference in the first and second scanning session, using dots moving coherently into one of eight (0°, 45°, 225°, 315°) randomly presented directions. Moving dot patterns were presented for 6 seconds followed by a variable inter-trial interval (ITI) of 9–12 seconds. A total of 24 trials per motion direction were obtained by randomly presenting each motion direction 4 times in 6 consecutive runs.

In the second experiment (i.e. V1 experiment) we localized the cortical representation of the lower right visual field. The responses were limited to a small visual field of view due to the limited coverage achievable with high resolution 3D GRASE. In both scanning sessions, participants viewed checkerboard stimuli in a traditional block design in either a target or a surround region (spatial frequency  = 0.6 cyc/deg, vertical screen size  = 20°, for further detail see [43]). The procedure consisted of alternating 12 s blocks of fixation or stimulation. Within each stimulation block, participants viewed contrast-reversing checkerboard stimuli (4 Hz) in the lower right visual field (target and surround), each presented on six occasions. The stimulated region began 0.5° (diagonally) from the center of fixation. The surround-area mapping stimulus comprised the inner 1° (diagonally from fixation). The target stimulus was further offset diagonally by 1° from the inner edge of the surround-area stimulus. Polar angle mapping in the first scanning session was used to distinguish between V1 and V2 activated regions supporting the placement of the measurement field of view in V1 for the second session (see below).

### MRI Acquisition

Data acquisition was performed at the Center for Magnetic Resonance Research (Minneapolis, MN, USA) using a 90 cm bore 7 Tesla whole-body magnet (Magnex Scientific, Abingdon, UK) driven by a Siemens console (Siemens Medical Systems, Erlangen, Germany) equipped with a high-performing head gradient insert. The RF coil consisted of a custom 6 channel receive array with elements (6 cm diameter) distributed symmetrically along the right - left direction and a separate open half-volume quadrature transmit coil to provide uniform excitation in visual areas. Such a design is especially ideal for high-resolution SE based fMRI because of the need for an efficient transmit profile that provides spatially uniform refocusing pulses and high sensitivity in the areas of interest. Furthermore the open design allows for presentation of visual stimuli over a large visual field of view.

The MT localization experiments (experiment 1, session 1) were conducted using a standard single shot gradient echo (GE) EPI sequence (echo time (TE)  = 15 ms, nominal flip angle (FA)  = 86°, slices  = 40,TR = 2000 ms) with a field of view (FOV) of: 128×128 mm^2^, and a 88×88 matrix resulting in a nominal resolution of 1.45×1.45×1.5 mm^3^.

For laminar response measurements with GE EPI in both V1 and MT (experiments 1 and 2, session 1) we used high resolution acquisitions [MT: (echo time (TE)  = 15 ms, maximum flip angle determined by a flip angle map  = 85°, slices  = 55, TR = 2000 ms, FOV = 128×128 mm^2^, matrix: 128×128, IPAT = 2, partial Fourier = 6/8, pixel bandwidth  = 1630, yielding a nominal resolution of 1×1×1 mm^3^)]; [V1: (echo time (TE)  = 17 ms, maximum flip angle determined by a flip angle map  = 85°, slices  = 38, TR = 2000 ms, FOV = 128×128 mm^2^, matrix: 160×160, IPAT = 2, partial Fourier = 6/8, pixel bandwidth  = 1375 yielding a nominal resolution of 0.8×0.8×0.8 mm^3^)].

In addition, in the second session of both experiments, we used a single-excitation, 3D gradient and spin echo (GRASE) sequence [37], [44] with restricted FOV achieved *via* inner volume selection [38]. To do this, following excitation of a 3D volume, a slab selective gradient (centric ordered phase encode) is applied along the phase encode direction during the train of 180 degree refocusing pulses, limiting the FOV in the phase encode direction, prior to 3D GRASE readouts [MT: (echo time (TE)  = 40 ms, TR = 2000 ms, FOV = 25.6×204.8×9.6 mm^3^, slice partial Fourier  = 5/8, pixel bandwidth  = 1955, matrix: 32×256×12, yielding a nominal resolution of 0.8×0.8×0.8 mm^3^)]; [V1: (echo time (TE)  = 40 ms, TR  = 2000 ms, FOV = 25.6×204.8×9.6 mm^3^, slice partial Fourier  = 5/8, pixel bandwidth  = 1955, matrix: 32×256×12, yielding a nominal resolution of 0.8×0.8×0.8 mm^3^)]. The RF train thus consisted of a 90 degree excitation pulse, followed by 8 refocusing pulses interleaved with EPI readouts (total echo train length  = 179 msec). Positioning of the limited FOV (phase encode and slice directions) 3D GRASE slab was optimally prescribed according to individual results of the localizer (MT experiment) or results of the preceding GE session (V1 experiment).

All scanning procedures started with a combined T_1_ weighted magnetization prepared rapid acquisition gradient echo (3D-MPRAGE) and proton density (PD) acquisition (176 slices, FOV = 136×256 mm^2^, matrix  =  136×256, voxel size  =  1×1×1 mm^3^). Because MR images exhibit large, undesirable signal intensity variations at high magnetic fields originating from heterogeneous RF coil profiles [45], [46], the additional gradient echo proton density images were acquired to compute a ratio image with the T_1_ dataset, reducing the bias field [45], [47]. The anatomical dataset was acquired for visualization of the functional results and analysis of cortical depth as well as laminar depth sampling.

### Functional data analysis

Functional images were analyzed using BrainVoyager QX 2.4.1 (Brain Innovation, Maastricht, The Netherlands) as well as custom code in MATLAB (The MATHWORKS Inc., Natick, MA, USA).

Preprocessing included: inter-scan slice-time correction (only for gradient echo data, since spin-echo data were acquired in 3D during a single echo-train of ∼200 msec), 3D rigid body motion correction, high-pass filtering (3 cycles) using a general linear model (GLM) Fourier basis set and a temporal gaussian smoothing with a full width half maximum (FWHM) kernel of 2 data-points. Average motion parameters for all six subjects were: 1) MT GE experiment: 0.03 mm±0.18 mm translation, 0.02°±0.15° rotation (mean ± SD of average parameters); 2) MT GRASE experiment: 0.09 mm±0.11 mm translation, 0.05°±0.05° rotation (mean ± SD of average parameters); 3) V1 GE experiment, 0.27 mm±0.20 mm translation, 0.07°±0.05° rotation (mean ± SD of average parameters); 4) V1 GRASE experiment, 0.25 mm±0.27 mm translation, 0.05°±0.28° rotation (mean ± SD of average parameters).

**Figure 1:**
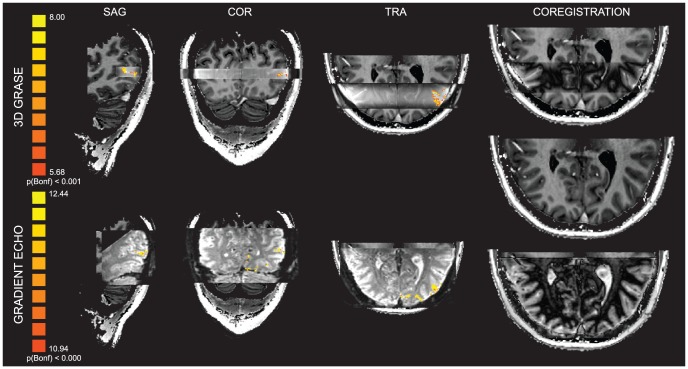
Individual coregistration results for 3D GRASE and GE-EPI. Activation maps (F-Maps sagittal, coronal and axial view) are superimposed to the functional volumes interleaved with the individual anatomical data. High-pass filtered functional volumes interleaved with the individual anatomical data highlight the correspondence between the data sets (last column axial view, middle represents native T1/PD for comparison).

Functional runs were co-registered to the individual anatomical data using the scanners positional information followed by a gradient driven alignment procedure as implemented in BrainVoyager QX. The results of the registration of functional and anatomical data were reviewed in each subject using information about the boundary between white and gray matter as extracted from the functional images. Figure 1 shows individual activation maps for the MT experiment (F-maps; sagittal, coronal and axial views) superimposed to the 3D GRASE and GE-EPI (merged with the individual anatomical data), in the last column the functional data are processed (high-pass filtered) and a difference image computed to highlight the coregistration results. This procedure was chosen since the native 3D GRASE data exhibits little anatomical contrast.

All statistical computations were performed on a single-subject level using a GLM with a linear predictor for each experimental condition convolved with a standard hemodynamic response function. In the three subjects participating in the MT experiment area hMT was defined using the localizer experiment (session 1) by selecting a region in the subjects unilateral posterior superior temporal sulcus that exhibited a significant (q [FDR] <0.05) response to bilateral and contralateral moving dot patterns in contrast to respective static patterns. To distinguish this area from the sub-region MST of the MT+ complex, voxels that did not exhibit a significant response to ipsilateral moving dot patterns were selected. Standard retinotopic mapping analysis was used to confirm the selected ROI's restricted response to the entire contralateral hemifield and exclude satellite motion selective regions [48]. For the second experiment the part of area V1 responding to the lower right visual field was defined based on retinotopic mapping (polar angle mapping only) and the GE target mapping experiment contrasting target and surround responses (q [FDR]<0.05).

The localizer procedure allowed the delineation of a region of interest (hMT and a defined part of V1) in each subject (from a total of six independent brain regions, three corresponding to hMT and three to V1). In two subjects we measured this from both ROIs as they participated in both experiments. Individual regions of interests (ROI) were used for the placement of the limited measurement field of view in the second (GRASE) scanning session and all subsequent analysis steps.

Within these regions of interest, overall percent signal change maps from the axis of motion (hMT) and flashing checkerboard (V1) data, were computed and sampled at different cortical depths (see below) together with cortical depth specific HRF responses. For the hMT experiment, the presented 8 motion directions were grouped into 4 pairs of opposing motion directions (axis of motion) to increase the selectivity of the voxels, since in monkeys opposing motion directions are known to be arranged in columns adjacent to each other [49]. This grouping was also performed to achieve a higher amount of trials per predictor to obtain more reliable parameter estimates. Axis of motion tuning was measured by fitting a standard GLM to the voxels' time-courses and extracting *t* values for every axis of motion in all subjects. The predictor with the highest fit, determined the characteristic preferred axis of motion direction for every voxel. Motion tuning curves were obtained by 500 fold cross validation (75% of data for preference classification, 25% of data averaging of voxel responses) of the above procedure in each relative cortical depth layer.

### Anatomical data analysis

In order to maintain unaltered resolution of the functional data while maximizing correspondence with the anatomical features, the ratio images (T_1_ over PD) were up-sampled to 0.8 mm isotropic resolution.

The detection of the white/gray matter boundary in the anatomical images was conducted with the automatic tools of BrainVoyager QX. To assure a correct definition of the boundaries in the regions of interest (V1 and hMT), all single subjects' segmentations were manually edited. After the definition of the white/gray matter boundary, the outer boundary (gray matter CSF) was automatically detected from the anatomical data. Additional manual editing was used to ensure the correct identification of this boundary in the regions of interests (V1 and hMT).

Cortical thickness was then measured with a procedure based on the Laplace equation [50]. Briefly, different constant “potential” (intensity) values were defined for the inner (e.g. 50) and outer (150) grey matter boundary and the grey matter voxels were initialized with the mean of these values (e.g. 100). While keeping the border values fixed, the intensity within the grey matter voxels is smoothed by replacing the intensity value of a voxel by the mean of the intensities in the 6-neighborhood. After 200 iterations the resulting solution of Laplace's partial differential equation implements a smooth transition of intensity values from one boundary to the other. From the obtained smooth field, a gradient value can be calculated at each voxel. Integrating along these gradient values results in “field lines” or “streamlines” used in cortical thickness calculations. For straight patches of cortex with a constant thickness, these streamlines correspond to straight lines connecting points at the WM/GM boundary with corresponding points at the WM/CSF boundary. For cortex patches with non-constant thickness, the resulting streamlines are slightly curved establishing a unique one-to-one mapping between corresponding points at the WM/GM boundary and the GM/CSF boundary.

The identification of *cortical depth dependent surfaces* was performed orthogonal to the thickness gradient in two orthogonal directions of constant depth using regularly spaced grid sample points. With a relative depth value of 0.5, for example, one grid axis will be created along a first chosen direction that traces a curved line through the middle of grey matter; at regular intervals along this first line, additional lines will be started that are orthogonal to the depth gradient and the first line, thus creating *regularly spaced* grid points along the second axis at the same relative depth level. At each sample point, the depth position is adjusted to ensure that the emerging curved two-dimensional surface is located at the desired relative depth level. It is also ensured that parallel lines keep a constant distance to each other and that lines moving in two orthogonal directions meet at a 90-degree angle. This procedure is performed for any desired relative depth level. As opposed to using irregularly spaced mesh vertices for depth calculation, this approach *provides precise geometrical information about path length and surface area* when following folded cortex at different relative depth levels. Furthermore, streamlines (created when following the gradient from one side of cortex to the other side) precisely relate corresponding points within sampled regular grids across multiple cortical depth planes. Importantly the definition of multiple surfaces within the same region of interest respects the local thickness of the cortex assuring corresponding points across surfaces at different depths to be *equally spaced*. Because of its dependence on cortical thickness the definition of cortical depth dependent surfaces as used here is limited to the cortical ribbon and does not grow outside the WM/GM boundary or the GM/CSF boundary.

**Figure 2:**
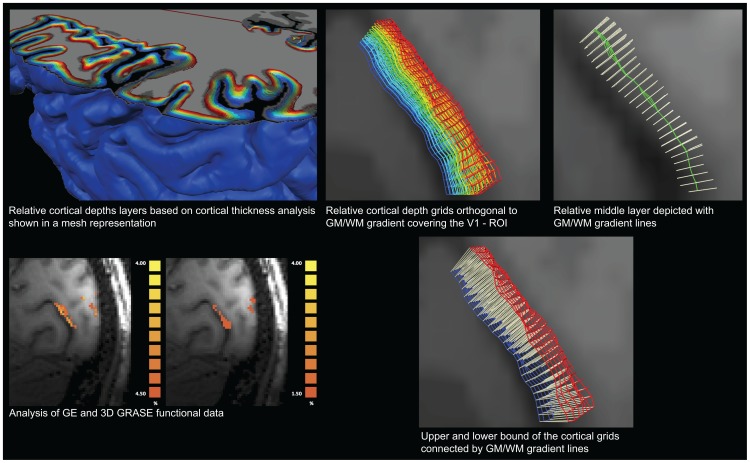
Cortical depth sampling procedure depicted in one exemplary subject for the analysis of cortical depth responses in area V1. The upper right corner illustrates our approach on a whole hemisphere, all analysis were performed using the local approach demonstrated to the right. A region of interest is defined based on the activation (F-map) as obtained by the analysis of the functional data (left columns, GE and 3D GRASE). Anatomical data is segmented and a measure of cortical depth obtained. Gridlines (color coded according to their relative depth) are constructed at relative depths (right column). Full grids, orthogonal gradient lines relative to the middle layer as well as the upper and lower bounds can be seen in the right column.

The approach is illustrated in figure 2 for the V1 experiment. The analysis starts with the definition of a region of interest based on the significant (q [FDR] <0.05; figure 1 bottom left) response of both GE and 3D GRASE data. The cortical thickness analysis procedure results in a map indicating the local cortical thickness. The local thickness information is used to compute cortical depth dependent surfaces (n = 9) within the region of interest or over the entire cortical hemisphere (figure 2 top left panel). Gradient lines can be defined connecting pairs of points across each surface (figure 2 top right and bottom right panels). Here we limited the analysis to pre-defined ROIs (V1 and hMT) since the activation is locally defined but the procedure can be applied to obtain surfaces across the whole cortical ribbon as highlighted in figure 2 (top left panel).

Using this novel approach, we sampled the cortex at 9 relative cortical depth levels spanning from 10% to 90% cortical thickness for the respective regions of interest and percent signal change maps for both experiments (V1 and hMT) were then sampled at these relative depth levels. It should be noted, that 9 relative cortical depth layers lead to an upsampled data representation of a factor of 1.5–2 considering an average cortical thickness of 3 mm and our functional data resolution.

## Results

In each individual subject the number of 0.8^3^ mm^3^ voxels defining the hMT area was approximately 500 (S1 left hMT, voxels  = 448; S2 right hMT, voxels  = 426; S3 left hMT, voxels  = 469) as well as approximately 240 for the analyzed part of V1 (S1 left V1, voxels  = 209; S2 left V1, voxels  = 239; S3 left V1, voxels  = 320).

**Figure 3:**
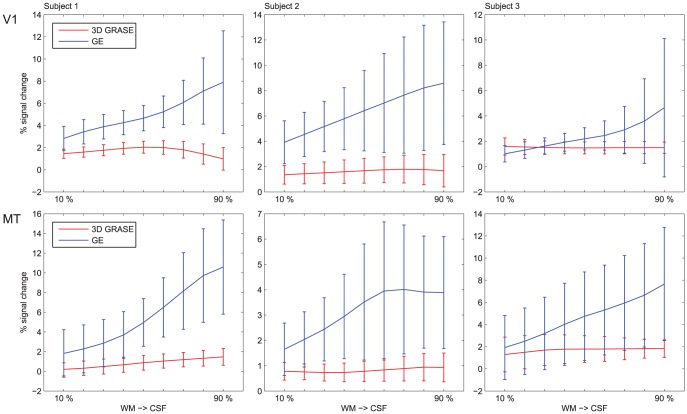
Single subjects' average percent signal cortical depth dependent signal in regions V1 (top) and hMT (bottom) for both GE-EPI (blue) and 3D GRASE (red). Error bars represent the standard error (across space) of the responses.

We examined the mean and variability (represented by the *standard error* of the signal over the depth dependent grid) of the percent signal change in regions hMT and V1 for both GE and 3D GRASE at different cortical depths (figure 3). Note that for both experiments the examined region exhibited a significant (q [FDR]<0.05) response to the stimuli. For both regions studied, an increase in percent signal change can be observed in the GE data when traversing the cortical sheet from the white matter to the cerebrospinal fluid boundary. It is noteworthy that GE is also characterized by increased spatial variability when moving from the white matter to the cerebrospinal fluid boundary. For the 3D GRASE data, the difference in the signal change across the depths is less evident and there was an overall lower percent signal change compared to the GE data. When comparing (repeated measures ANOVA) the slope of the two curves, we observed a significantly (p<0.01) higher slope for GE compared to 3D GRASE. A greenhouse-geisser corrected repeated measures ANOVA revealed no difference in relative cortical depth values for 3D GRASE (F = 2.012, p = 0.197) but a significant main effect of relative cortical depth for GE (F = 23.999, p = 0.003). Examining the main effects, a linear fit of relative cortical depth provides the best approximation to the data for GE (F = 27.446, p = 0.003) while linear as well as higher order functions remain not significant for 3D GRASE indicating a significantly larger contribution from pial vessels for GE when moving towards the CSF.

**Figure 4:**
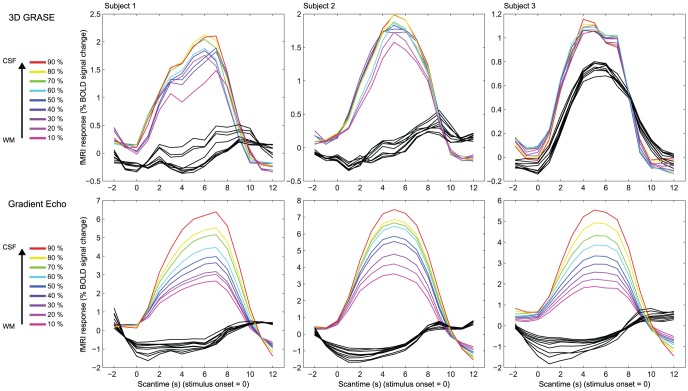
Single subjects' event related average of the response to flashing checkerboards in area V1 as obtained with 3D GRASE (top) and GE-EPI (bottom). Colored lines represent the response to the stimulated quadrant (color corresponding to the relative cortical depth of the response). Black lines represent the response to the portion of visual field of view surrounding the stimulated region.

For the V1 experiment, where only a single stimulus type was used and fixation was always presented between stimuli, we computed event related averages around the stimulus onset. Figure 4 depicts the results of the event related averages plotted for each layer (target response in colored lines and surround response in black lines). A clear differentiation in the amplitude of the event related averages is observed in the GE data with the highest peak near the cerebrospinal fluid and the lowest peak near the white matter boundary. In the 3D GRASE data, all sampled depths showed a similar averaged event related functional response with no systematic layer specific differences. The presence of a response to the surround stimulus in the targeted region for the 3D GRASE data of one subject (subject 3) can be explained by an erroneous placement of the measurement FOV in this specific subject, owing to the precision required when having to acquire data from limited field of views.

**Figure 5:**
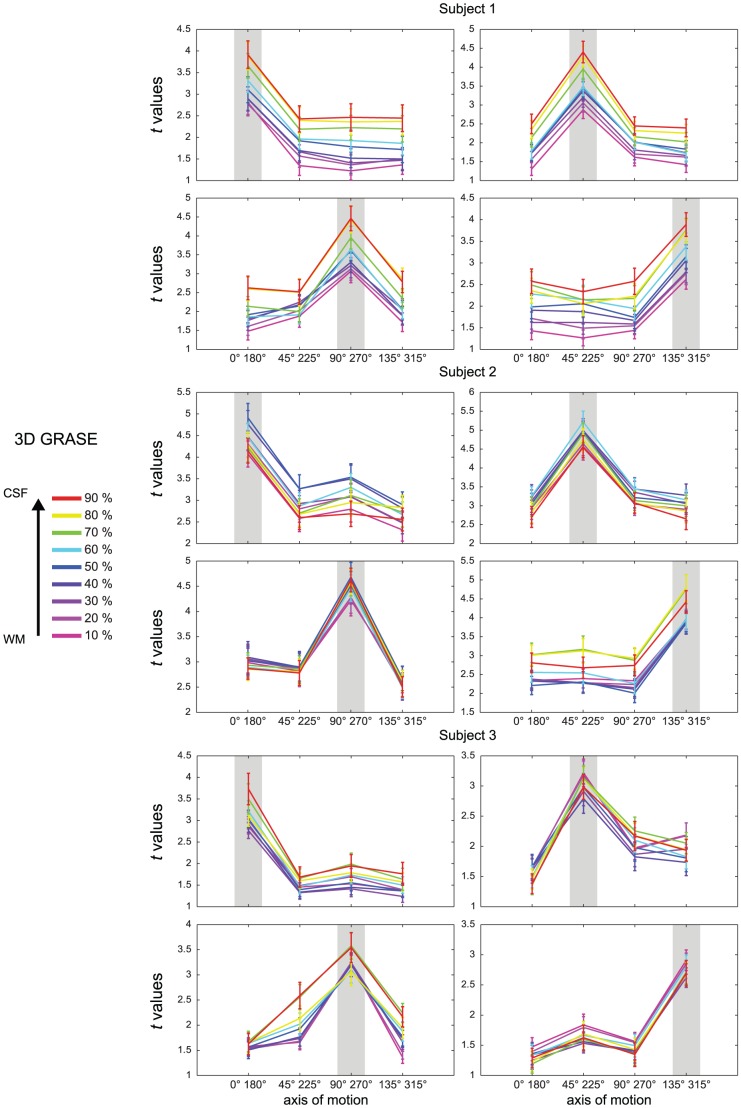
Single subjects' tuning curves (computed in cross validation n = 500) of the responses to different axis of motions stimuli in area hMT for 3D GRASE. Error bars represent the variability (standard error) across the splits. Colors represent different relative cortical depths. Each quadrant shows the tuning curves to one axis of motion.

**Figure 6:**
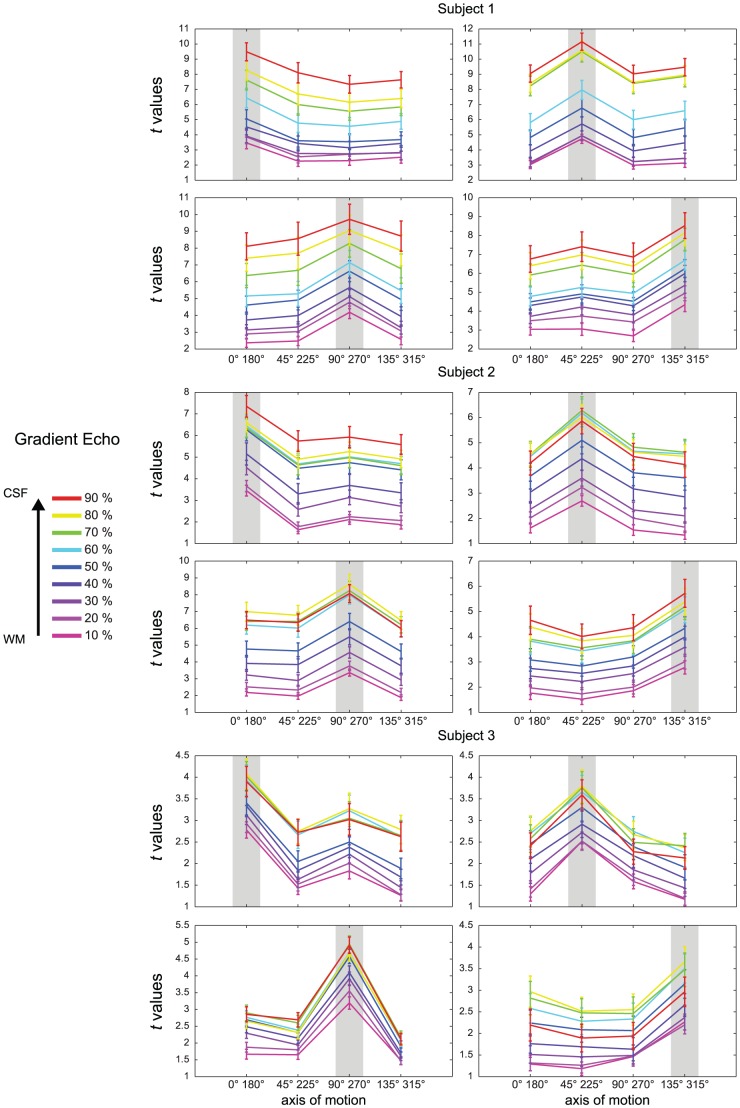
Single subjects' tuning curves (computed in cross validation n = 500) of the responses to different axis of motions stimuli in area hMT for GE-EPI (bottom). Error bars represent the variability (standard error) across the splits. Colors represent different relative cortical depths. Each quadrant shows the tuning curves to one axis of motion.

**Figure 7:**
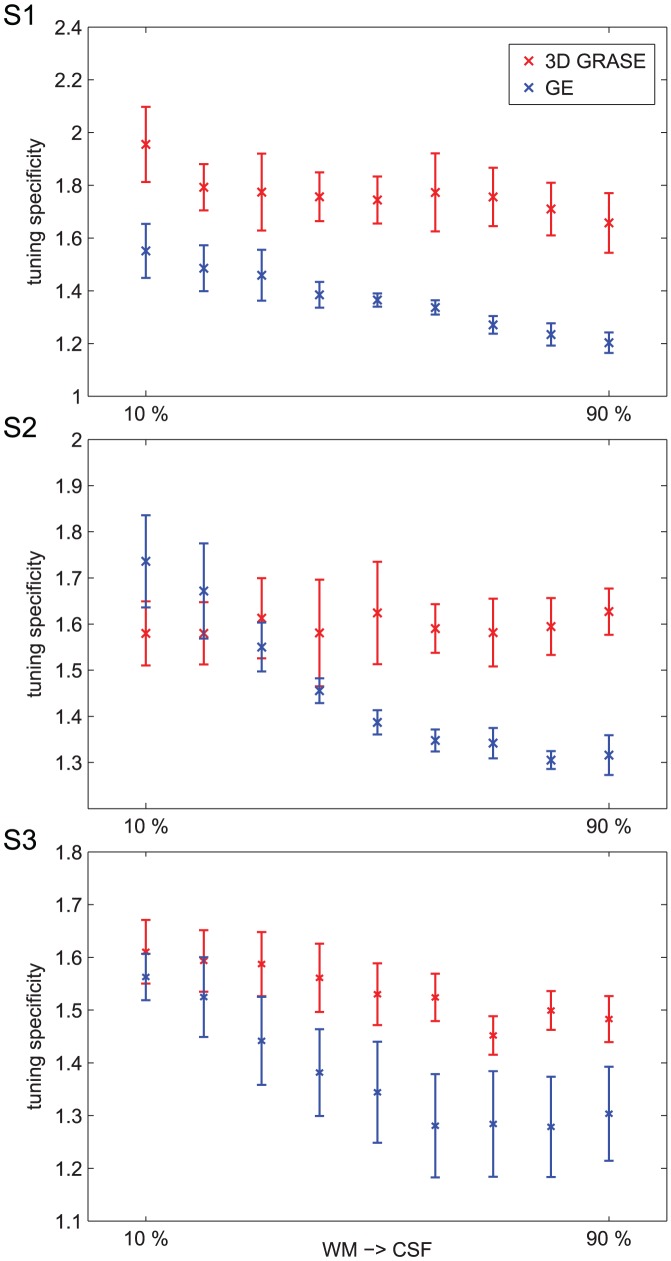
Single subjects' specificity of the responses to the preferred axis of motion in area hMT for both 3D GRASE (red) and GE-EPI (blue). Error bars represent the variability (standard error) across the different tuning directions. Specificity is computed as the ratio between the preferred response and the average response to the non-preferred directions. Error bars represent standard errors across voxels.

**Figure 8:**
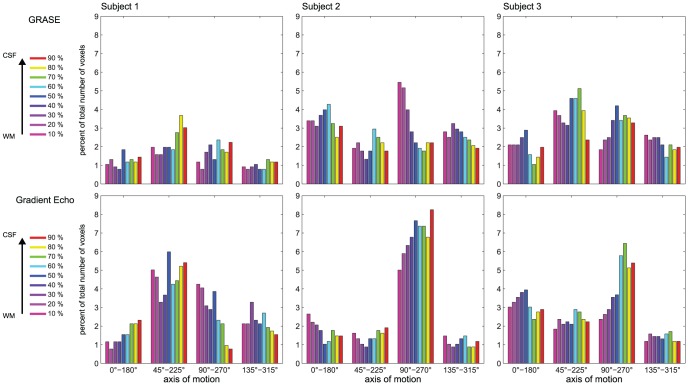
Single subjects' results representing the cortical depth dependent percentage of voxels tuned to each axis of motion in area hMT for both 3D GRASE (top) and GE-EPI (bottom). Colors represent relative cortical depths.

The analysis outlined in figure 3 represents the cortical depth dependent variability of the response to a single stimulus. To evaluate the specificity of the fMRI responses across layers obtained with the different sequences, we computed axis of motion tuning curves for area hMT using a cross-validation approach [10]. For all subjects, (figure 5 and figure 6) the tuning curves obtained using 3D GRASE were more similar across the cortical depths while these same profiles diverge in the GE data (note the response increase towards the surface; error bars represent the variability (standard error) across the splits). Importantly, tuning characteristics for the specific axis of motion (gray highlighting, figure 5 and figure 6) show a more sharply tuned curve for 3D GRASE when compared to GE data especially but not limited to the superficial layers. A tuning specificity index was computed by calculating the ratio of the response for the labeled axes of motion divided by the mean response of the layer towards all other axes of motion. The results of the tuning specificity index can be seen in figure 7 (error bars represent the variability (standard error of the mean) across the different tuning directions). With the 3D GRASE data, no significant difference in the tuning specificity can be observed across the sampled relative cortical depths. However, in all subjects' GE data, tuning specificity decreases from the white matter to the CSF boundary. Testing for the difference between GE and 3D GRASE across all layers and subjects resulted in 3D GRASE having significantly (F = 20.878, p<0.05) higher specificity than GE, a result consistent with some depth dependent contamination of the BOLD point spread function from surface vessels. Note that the effect progresses when moving towards the CSF indicating that the contamination from surface vessels spreads well inside the cortical ribbon. No significant increase in tuning specificity could be observed in the middle layers for either the GE or 3D GRASE data. We also measured the percentage of voxels tuned to a given axis of motion direction as a function of the cortical depth. This analysis, reported in figure 8 for all subjects, shows a bias of the GE measurements for a single axis of motion, which is disproportionately represented compared to other axes, and is not present in the 3D GRASE data.

## Discussion

In this study, subjects underwent 7T fMRI with high isotropic spatial resolution using both conventional GE-EPI and T_2_ weighted 3D GRASE. Depth dependent functional responses from two different cortical regions (hMT and V1) were compared between the two sequences to ascertain whether 3D GRASE with inner volume excitation, which allows (over limited field of views) for more efficient acquisitions compared to 2D SE with inner volume excitation, provides any specificity advantages over GE for layer specific fMRI studies in humans.

### Cortical depth sampling procedure

Several approaches have been used to define sampling directions or surfaces for the cortical depth dependent analysis of human fMRI responses. Sampling lines, defined orthogonal to the white/gray matter or gray-matter/CSF boundary [23], [24], do not respect the local curvature of the cortex. Alternatively the use of surfaces evolving from the white/gray matter boundary to the gray-matter/CSF boundary has been proposed [21], [22], [51]. While also being able to take into account the local thickness of the cortex, these approaches may result in irregular sampling within each depth due to the varying distance between vertices in each surface. Here we used a local approach that, while considering the local curvature and thickness of the cortex, results in a set of surfaces with regularly spaced grid points within the cortex that allow one to regularly sample functional data at each relative depth level. It has to be noted that this technique does not guarantee homogeneous sampling of the same cortical layer (defined by neuronal cytoarchitecture) throughout the analyzed regions [52], [53]. It has been reported, for example, that the position of cortical layer IV varies with respect to the cortical depth between gyri and sulci [54]. To deal with this potential problem, it has been shown that the T_2_* in the middle layers is shorter [24], [47], [55] and can be used as an anatomical constraint to better align the layer profiles sampled over different locations and through different orientations [24]. A dark ‘band’ of signal intensity in V1 in the middle of the gray matter (i.e. line of Gennari) was not reliably identifiable in our EPI data, possibly due to limited spatial resolution (0.8 mm) or contrast. As such, we feel that using an approach like this in our data would result in errors, likely due to significant partial volume effects in the local T_2_* values. Further, even if such anatomical information could be reliably identified in V1, it does not apply to other visual areas, such as hMT, as studied here. Furthermore, while the anatomical information present in the T_2_* contrast is valuable, we feel that separate anatomical data should be used for the identification of intra-cortical landmarks in order to avoid potential “double dipping” [56]. Here, when possible, to be extremely conservative, we limited the analysis to relatively *straight* portions of cortex since we have no means of calculating an error in displacement due to the lack of real ground truth cytoarchitectonic data.

### The use of GE imaging for layer specific fMRI vs. T_2_ weighted approaches

T_2_ weighted signals have been suggested to be advantageous for sampling cortical depth dependent functional responses because of the reduced sensitivity to large (superficial) draining veins [12]–[15], provided the T_2_* contamination is not too large [14]. Despite potential advantages, the use of high-resolution 2D SE-EPI sequences is limited by excessive power deposition and non-uniform B_1_ profiles at high fields, which limits the available SNR, making an approach as was used here for 2D GE (large FOV) imaging (i.e. high resolution imaging with in plane parallel imaging accelerations), not practical with the coils used in this study. Further, the use of 2D inner volume excitation, which allows arbitrary reduction of the phase FOV, eliminating the need for in plane accelerations with subsequent SNR losses, due to cross irradiation of spins in the slice, acquiring more than a single slice is temporal inefficient. To deal with this, thick single slice studies and relatively straight portions of cortex have been used [8], [9]. Using a single slice approach would not sample the cortex sufficiently or provide enough voxels to allow reliable measurements of layer dependent functional responses. As such, with the available technology, 2D-SE EPI with a full FOV or with reduced FOVs is not a feasible approach for layer specific investigations as employed here. Recent applications of 3D GRASE with inner-volume selection [10], [25], as used here, have shown that it provides an efficient alternative to previous high-resolution 2D SE-EPI techniques, by allowing sampling of the complex curvature of the human cortex with high isotropic resolution along with columnar level specificity. However, the functional contrast in 3D GRASE images consists of both T_2_ and stimulated echo weightings. It has been shown with modeling that the contrast generated by stimulated echoes originates from dynamic averaging due to diffusion and is thus similar to conventional SE-EPI with the potential advantage of higher signal changes due to longer sampling periods [39]. However, whether or not 3D GRASE provides any specificity advantages over conventional GE for high resolution applications of cortical layers or columns, as has been shown for conventional SE BOLD imaging at high fields, has not yet been established. Here we demonstrate that mean functional responses obtained with 3D GRASE, while exhibiting the expected decrease in functional sensitivity compared to GE (characterized by overall lower percent signal changes), are not biased towards the surface (figure 3), as are the GE data. Due to limited SNR in the MT ROIs (due to the need for in plane GRAPPA and the coil geometries), the single shot full FOV GE-EPI acquisition in the MT experiment was limited to a resolution of 1 mm isotropic. The limited field of view obtained with the 3D GRASE and inner volume excitation, allowed acquiring data in the same region without the use of GRAPPA acceleration. This, together with the 3D readout resulted in sufficient SNR in our 0.8 mm isotropic 3D GRASE acquisitions. It has to be noted that the due to the signal decay during the 3D readout train of GRASE, blurring is introduced in the slice direction (i.e. decreasing the resolution in the z-direction). We estimate this effect, based on measurements of the signal decay during the readout period [57], to be on the order of 20% for the readout train length used here, resulting in a resolution of about 1 mm in the slice direction. This effect did not result in significant blurring of the activation as it can be noted in figure 1 for an axial acquisition. The curvature of the cortical layers and their angle with respect to slice orientation, results in minimizing the effect of slice blurring on the cortical depth dependent profiles of 3D GRASE and their overall trend, which indicates a reduced bias towards the surface.

Additionally, we show that the specificity of the response (measured as the sharpness of the tuning curves) is generally higher for 3D GRASE than GE (figure 7) despite a larger overall response in GE, consistent with columnar specificity measurements in human ODCs [8]. Further, the non-specific superficial draining veins appear to deteriorate the quality of the responses of GE signal towards the surface, while the specificity of 3D GRASE remains unaltered throughout the cortex (figure 7). It has to be noted that this effect, while larger on the surface, is not limited to the superficial layer. The decreased specificity of the GE signal is confirmed by the analysis reported in figure 8 showing a bias towards a single axis of motion throughout the cortical depths for GE data. This could be explained as an effect introduced by a large vessel carrying specific functional information [58] (i.e. specificity to one axis of motion orientation) as previously shown in GE-EPI acquisitions [8].

### Layer response profile and sensitivity to cortical layer IV

In both human MT and V1 our cortical sampling results show a significantly higher slope (increase in the functional response) towards the surface of the cortex for GE compared to 3D GRASE. This observation is in agreement with previous animal and human studies [12]–[14], [21]–[24], [59]. Our cortical depth analysis did not reveal any significant increases near the middle of the cortex in GE or GRASE data (i.e. suggestive of no greater responses for cortical layer IV compared to nearby layers) in either V1 or hMT. This finding is in agreement with that of Polimeni and colleagues [22]. However, there are differences between our findings and other previous human reports [21], [23], [24], which showed small signal increases near cortical layer IV. This could be explained by different normalization procedures of the fMRI images, different cortical sampling procedures or the fact that the previously reported signal increases are quite small compared to the much larger surface effect. Note that in the V1 analysis, when possible, we restricted sampling to a straight region of the cortex, limiting the possible anatomical variability of the position of cortical layer IV with respect to the cortical depth. It should also be noted that our data were acquired at relatively short TEs (17 ms), so the lack of a middle layer ‘bump’ is not due to the TE chosen [24]. Importantly, our obtained tuning specificity measures may differ when using significantly different TEs. Finally, we used flashing checkerboards in a limited portion of the visual field of view for V1 measurements, and moving dots for hMT measurements. As such, differences in stimulus properties could explain differences in the literature on whether a stronger signal in the middle layers is observed; more studies are, however, needed to support this hypothesis.

The existence or lack of a small increase in signal at the middle of the cortex, however, says nothing about the ability of fMRI to disentangle layer specific functional information. In fact, using a similar 3D GRASE acquisition protocol, significant increases at the middle layers were not reliably detected either, while differential profiles provided valuable information regarding underlying neuronal processes regarding object recognition [25]. Also of note, is that this study employed different cortical depth sampling tools, and a different visual paradigm to elicit functional responses. The magnitude of the signal increase at the middle of the cortex, as has been reported in humans [21], [24], is quite small (three times smaller) compared to effects from the surface, which may likely be the more limiting factor in the resolvability of layer specific neuronal information with fMRI. The extent to which signals from the surface spread through the cortical layers (i.e. BOLD extravascular point spread) may in some cases be on the order of the signals from the middle layers, making the reduction of surface effects as important as enhanced middle layer effects.

## Conclusions

We have investigated the possibility and advantages of using T_2_ weighted BOLD contrast as obtained with the 3D GRASE sequence to measure cortical depth dependent fMRI responses in limited field of views. In order to adjust to varying cortical thickness, the high isotropic resolution functional data were sampled using regularly spaced two-dimensional grids at selected relative cortical depth levels. Our GE measurements confirm previous reports of significant superficial large draining veins effects. This effect was significantly reduced with 3D GRASE, which also exhibited higher specificity, making it an attractive alternative for localized layer specific fMRI studies in humans.
